# Applying Movement Constraints to BLE RSSI-Based Indoor Positioning for Extracting Valid Semantic Trajectories

**DOI:** 10.3390/s20020527

**Published:** 2020-01-17

**Authors:** Hani Ramadhan, Yoga Yustiawan, Joonho Kwon

**Affiliations:** 1 Department of Big Data, Pusan National University, Busan 46241, Korea; hani042@pusan.ac.kr (H.R.); yoga017@pusan.ac.kr (Y.Y.); 2 School of Computer Science and Engineering, Pusan National University, Busan 46241, Korea

**Keywords:** indoor positioning, BLE RSSI data, internet of things, semantic trajectory

## Abstract

Indoor positioning techniques, owing to received signal strength indicator (RSSI)-based sensors, can provide useful trajectory-based services. These services include user movement analytics, next-to-visit recommendation, and hotspot detection. However, the value of RSSI is often disturbed due to obstacles in indoor environment, such as doors, walls, and furnitures. Therefore, many indoor positioning techniques still extract an invalid trajectory from the disturbed RSSI. An invalid trajectory contains distant or impossible consecutive positions within a short time, which is unlikely in a real-world scenario. In this study, we enhanced indoor positioning techniques with movement constraints on BLE (Bluetooth Low Energy) RSSI data to prevent an invalid semantic indoor trajectory. The movement constraints ensure that a predicted semantic position cannot be far apart from the previous position. Furthermore, we can extend any indoor positioning technique using these movement constraints. We conducted comprehensive experimental studies on real BLE RSSI datasets from various indoor environment scenarios. The experimental results demonstrated that the proposed approach effectively extracts valid indoor semantic trajectories from the RSSI data.

## 1. Introduction

Indoor location-based services have given rise to the requirement of establishing various safety measures in IoT (Internet of Things)-enabled smart buildings in recent years. Indoor trajectories [1] support these services for several applications, such as people behavior analytics, movement patterns extraction, next-to-visit recommendations, and hotspot detection. Positioning devices, such as Wi-Fi [2], BLE (Bluetooth Low Energy) [3,4,5,6,7,8], and RFID (radio frequency identification) [9], provide the trajectories using indoor positioning techniques.

Indoor trajectory is a sequence of paired timestamps and visited positions of a user in indoor spaces. To capture these positions, the users hold a device, such as a smartphone, that receives the RSSI (received strength signal indicator) of the positioning devices in the indoor space while walking. However, the RSSI is often missing or unstable due to delayed transmission, interference from the walls, and clashing signals. Thus, we require a technique called indoor positioning to estimate the user’s correct position.

Indoor positioning methods usually require a dataset of collected RSSI data and the associated semantic position. This dataset is called the reference set. Then, the methods estimate the current user position using the knowledge from the reference set. Some popular indoor positioning techniques are the Hidden Markov Model (HMM) [4,5,9,10,11,12], k-nearest neighbors (kNN) [6,7,8], and Deep Neural Networks (DNN) [3,13]. Those methods (HMM, kNN, and DNN) utilize machine learning as their core. HMM and DNN methods learn the behaviour of the trajectory from the reference set. Thus, they can predict the next position after the current position estimation given the next observation of RSSI data. In contrast, kNN methods estimate a position by comparing the difference of current RSSI observation to the reference set. The kNN method decides the current RSSI’s semantic position such that the semantic position’s RSSI in the reference set has the smallest difference with the current RSSI. However, HMM, kNN, and DNN still cause errors in estimating the user’s position that leads to inaccurate positioning due to missing and unstable RSSI data.

Erroneous indoor positioning can give rise to invalid trajectories, which contains at least one impossible movement between a pair of consecutive positions. Suppose that an indoor positioning technique mispredicts the current position to be in the next room, which is impossible because a wall separates the previous position, while the room and the room’s door are on the farther side. Another case of erroneous indoor positioning is the “jumpy” transition due to the missing and unstable RSSI data. Hence, the estimation of the current position is significantly deflected from the previous position. Thus, we would like to adopt the contextual outdoor area definition [14,15,16] to incorporate the structure of the indoor space. We call this newly defined area semantic position.

The semantic positions, instead of conventional 2D positions, are useful for several use cases, such as real-time next-to-visit recommendation service. It is more understandable for users to check a recommendation in semantic positions, such as Next-to-visit: from Room-History, after Corridor turn right, instead of 2D positions unless visualized by some indoor map. This case also challenges indoor positioning to work in real time, which means that the most likely position has to be estimated without seeing the full trajectory, similar to incremental map-matching [4,11]. We can build these real-time recommendations based on extracting real-time semantic trajectories.

A semantic trajectory describes the sequence of visited semantic position instead of the usual indoor 2D positions. This 2D position is not understandable until visualized. To acquire this semantic trajectory in an indoor space, we can simply get the semantic positions by associating certain areas in 2D space as semantic locations.

**Figure 1:**
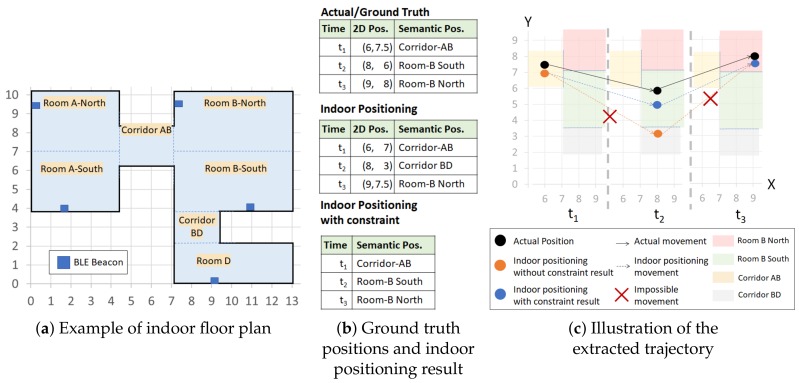
Example of indoor positioning and trajectory extraction

**Example** **1.** *Suppose an indoor floor plan similar to Figure 1a. From the positioning result in Figure 1b, we see an extracted 2D indoor trajectory that denotes the movements of a user from position (6,7) at $t_{1}$ to position (8,3) at $t_{2}$ and then to position (9,7.5) at $t_{3}$ in a short time in Figure 1c. Thus, if we know that position (6,7) is at the Corridor AB, position (8,3) is at Corridor-BD, and position (9,7.5) is at Room B-North, we can define a more understandable trajectory such as Corridor AB → Corridor BD → Room B-North.*

Although we can translate the 2D positioning to semantic positions using a type of mapping technique, due to the erroneous indoor positioning techniques, we should consider a way to prevent the extraction of invalid trajectories. An invalid trajectory contains a distant or an impossible displacement, such as moving directly from a room to another room separated by wall without a door. Thus, we introduce movement constraints to restrict an indoor positioning technique to only infer a position that is close and not obstructed from previous positions.

**Example** **2.** *In Figure 1b, because of incorrect positioning, it is impossible to move from Corridor AB to Corridor BD as depicted in Figure 1c. Therefore, the extracted trajectory is an invalid trajectory because it contains an impossible movement. This impractical movement, however, can be restricted if we define a movement constraint: we can only go to Room B-South after Corridor AB. Then, using the movement constraint, we can get a valid trajectory Corridor AB → Room-B-South → Room B-North as depicted in Figure 1c.*

In this study, we applied movement constraints in RSSI-based indoor semantic positioning and trajectory extraction. Considering the time interval of each inferred position, which should be short, consecutive positions could not be far apart or blocked by an obstacle. Thus, the movement constraint can be used to prevent the occurence of far or impossible consecutive positions. Therefore, we can guarantee the validity of the extracted trajectory.

The indoor positioning techniques can apply the movement constraints to estimate the current semantic position of a user. We can extend HMM to apply the movement constraints as state transitions, considering the semantic positions as the states. Meanwhile, kNN can be extended with the movement constraints to reduce the search space of current semantic position prediction only to nearby semantic positions by analyzing previously estimated semantic positions. Similar to kNN, other machine learning approaches, such as neural networks, can work as indoor positioning techniques and can prevent the occurence of invalid trajectories by limiting the range of outputs to nearby consecutive positions.

We performed experiments using three real datasets of people moving inside a building using BLE beacons as RSSI-based positioning devices. To tackle incorrect indoor positioning, we performed sliding-window aggregation to reduce the the instability and incompleteness of BLE RSSI data. Different scenarios for semantic position definitions and beacon deployment were considered. To measure the validity and quality of the approach, we devised several metrics for semantic positions with some resemblance to 2D setting.

The key contributions of this paper can be summarized as follows.

- We adopt movement constraints to machine learning-based indoor positioning methods, such as kNN, HMM, and neural networks, to extract valid semantic trajectories in real time from the BLE beacons RSSI in indoor environment.
- We presented a detailed experimental evaluation for comparing our approach with current state-of-the-art approaches for different environments. The experimental results demonstrate the performance benefits and feasibility setting of the proposed constrained approach.

The rest of this paper is organized as follows. Section 2 provides the related research. Then, we continue to explain the basic knowledge of this work in Section 3. Section 4 describes the proposed constrained approach using several indoor positioning techniques. Section 5 presents the details of the experiment design, the results, and the discussions. Finally, we conclude this paper in Section 6.

## 2. Related Works

In this section, we briefly review some related works and provide a comparison to the proposed method.

### 2.1. RSSI-Based Indoor Positioning Techniques

Indoor positioning yields the position of a current user from given several measurements. Some techniques utilize inertial sensors [4,17] and RSSI-based sensors [4,5,7,8,13,17] in an indoor environment. However, most of them work on 2D exact positions, such as particle filter [17], kNN [7,8], and reinforcement learning [13]. Another approach [3] directly infers the room where a user resides by performing classification using convolutional neural network on transformed images that consider BLE signals and positions as feature. However, only a few methods [13,17] along with the HMM method [4,5] consider the trajectory as the output of indoor positioning. Not dealing with this issue leads to invalid trajectory, as the consecutive positioning result may be far apart.

### 2.2. Indoor Trajectory Extraction

Unlike the outdoors, the indoor environment may have a different sense of semantic position and trajectory. Indoor semantic positions tend to have smaller coverage and finer information, e.g., toilet or hallway, unlike outdoor semantic positions, e.g., restaurant or office. One of the representations of the movements on an outdoor semantic trajectory is the road-network [18]. The vehicles move in accordance to the road network as they cannot trespass a building in a normal situation, e.g., not breaking any traffic laws. However, in the indoor environment, we cannot restrict the movement to only the passages and corridors. A large indoor space can contain several semantic positions owing to the specific definition of the area, e.g., a hall can contain numerous exhibition objects and we would like to identify a semantic position as the area around an object. Thus, constructing a semantic trajectory in an indoor environment is different from that in the outdoor environment.

The semantic indoor trajectory extraction process is similar to RFID cleansing [9,19,20]. Similar to Reference [9], we do not assume that all detailed characteristics or spatiotemporal constraints of the BLE beacons in the indoor environment are known. This case is different from previous works [5,19,20] where a position was directly related to a beacon. According to Reference [9], the learning-based method cleans the RFID of the trajectory data to an RFID observation at a time. In our case, we may capture the RSSI signals from different beacons or miss the signal completely at some semantic positions. Thus, we leverage the machine learning-based approach to infer the semantic location from the overlapping and missing observations.

In contrast, we consider a case of indoor semantic trajectory extraction similar to outdoor map-matching [11,12], especially in road networks. Most of these cases use incremental HMM, which takes the distance directly as the observation (GPS data). In indoor cases, we can compute the distance [10] or directly use the existence of captured RSSI in the current position [5] as observation, even though the RSSI is incomplete and noisy. However, the number of deployed beacons in such a setting is usually large. Previous works rarely studied map-matching using RSSI data in different environments, such as sparsely deployed beacons.

Moreover, the use of non-incremental map-matching techniques in indoor semantic positions [5] ensures that the user moves in a valid trajectory and outputs optimal result. However, it needs to see the full trajectory. This style is not applicable in a real-time trajectory extraction case. In contrast, despite yielding suboptimal results, the incremental map-matching techniques work in real time and may suffer a “ping-pong” effect [12], similar to invalid movements. Several online HMMs have attempted to solve the issue. The bounded variable sliding window (BVSW) approach on the online viterbi algorithm [11] solves this issue in outdoor environment, while the local HMM [4] estimates the 2D position using Wi-Fi signal in the indoor case. However, the local HMM still suffers from some invalid movements.

To prevent this issue, we apply movement constraints on machine learning (ML)-based RSSI indoor positioning. We aim to predict the next position that complies with the real-world situation that should not be too far from the current position. These constraints apply to any machine learning-based indoor positioning method, with special cases for HMM and kNN. The constraints reflect the transition probability in HMM and reduce the search space for kNN. This approach also applies to different scenarios of beacon deployment in the indoor environment. Thus, based on this approach, we can extract a valid indoor semantic trajectory, which has been demonstrated through the experiment result.

## 3. Preliminaries

In this section, we present the problem definition in Section 3.1, notions of the input of BLE RSSI sequences in Section 3.2, and the notions of indoor semantic trajectory in Section 3.3.

### 3.1. Problem Definition

**Figure 2:**
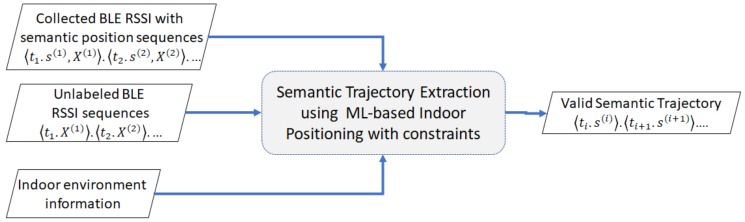
Problem definition to extract valid trajectories.

Our problem, depicted in Figure 2, is described as follows. We need to extract valid trajectories from the input of unlabeled BLE RSSI sequences in an indoor environment while the user is moving. To perform this, we use the ML-based indoor positioning with constraints that learns the characteristic of the BLE RSSI sequences from the previously collected BLE RSSI dataset with semantic position sequences as labels. The movement constraints come from the indoor floor plan, which represents the information of the indoor environment. These movement constraints are represented by a semantic graph in later parts.

### 3.2. BLE RSSI Input

When a user is moving in an indoor environment with BLE beacons, his/her RSSI capturing device, e.g., smartphone, captures a collection of RSSI observations from the beacons according to his/her position at a time. A raw RSSI has a negative integer value with a maximum value of −1. If a capturing device is closer to a beacon, it will capture the higher RSSI value. A capturing device may capture a weaker signal or even miss a signal due to several circumstances, e.g., delayed transmission, beacon antenna’s orientation, obstructing objects, and overlapping signals. These circumstances may inform us about the surroundings of the indoor space, such as disturbance from many objects or deflected by walls.

Suppose the indoor environment contains *M* deployed beacons that emit *M* RSSI values. Then, we can represent these *M* RSSI values as a vector.

**Definition** **1.** *An RSSI vector $X^{\left(t\right)}=\{x_{1}^{\left(t\right)},x_{2}^{\left(t\right)},$$...,x_{M}^{\left(t\right)}\}$ consists of M elements of negative integer and 0 values that indicate the signal strength of the observed RSSI at time t. An element $x_{m}^{\left(t\right)}$ that equals 0 indicates that, at time t, the RSSI observing device misses the RSSI of beacon m. The higher value of $x_{m}^{\left(t\right)}$ (except 0) indicates that the position beacon m is closer to the RSSI observing device.*

Suppose we have collected BLE RSSI observations previously knowing our semantic position $s^{\left(t\right)}$ at each time *t*. Then, we have several sequences of BLE RSSI observations paired with semantic positions. We call this collected sequence reference set *R*.

**Definition** **2.** *A sequence of RSSI observations T consists of |T| RSSI vectors at timestamp t. Thus, we can define T as $T=<\left(t_{1},X^{\left(1\right)}\right),\left(t_{2},X^{\left(2\right)}\right),...,\left(t_{\left|T\right|},X^{\left(|T|\right)}\right)>$.*

**Definition** **3.** *A sequence of RSSI observations with semantic position $T^{\prime }$ consists of $|T^{\prime }|$ RSSI vectors where each vector is attached with a semantic position $s^{\left(t\right)}$ at timestamp t. Thus, we can define $T^{\prime }$ as $T^{\prime }=<\left(t_{1},X^{\left(1\right)},s^{\left(1\right)}\right),\left(t_{2},X^{\left(2\right)},s^{\left(2\right)}\right),...,\left(t_{\left|T\right|},X^{\left(|T^{\prime }|\right)},s^{\left(|T^{\prime }|\right)}\right)>$.*

**Definition** **4.** *A sequential reference set R is a set that contains sequences of RSSI vectors with semantic positions. We define this set as $R=\{T_{1}^{\prime },$$T_{2}^{\prime },...,T_{\left|R\right|}^{\prime }\}$, where |R| denotes the number of the collected sequences.*

The sequential relationship is useful for a sequential-based indoor positioning method, such as Hidden Markov Model. Sometimes, we omit the sequential relationship between timestamps for some indoor positioning techniques, such as kNN or deep neural network. Thus, we drop the timestamp information $t_{i}$ of the reference set *R* and define another form of reference set $R^{\prime }$.

**Definition** **5.** *A nonsequential reference set $R^{\prime }$ is the set of RSSI vectors paired with semantic positions without sequential relationship. Thus, $R^{\prime }=\left\{\left(X^{\left(1\right)},s^{\left(1\right)}\right),\left(X^{\left(2\right)},s^{\left(2\right)}\right),...,\left(X^{\left(|R^{\prime }|\right)},s^{\left(|R^{\prime }|\right)}\right)\right\}$, where $|R^{\prime }|$ denotes the number of collected RSSI vectors.*

**Figure 3:**
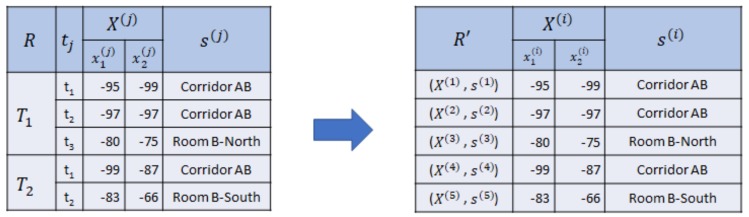
Transforming *R* **(left)** to $R^{\prime }$ (**right**).

**Example** **3.** *In Figure 3, we collect two sequences of RSSI observations with semantic position $T_{1}^{\prime }$ and $T_{2}^{\prime }$ using two beacons (M=2) as reference set R, where $T_{1}^{\prime }$ consists of three RSSI vectors and $T_{2}^{\prime }$ consists of two RSSI vectors. Then, we drop the sequential relationship, symbolized by the timestamp $t_{j}$ and sequence $T^{\prime }$. Hence, we acquire $R^{\prime }=\{\left(X^{\left(i\right)},s^{\left(i\right)}\right)\left|1\leq i\leq \right|R^{\prime }\left|\right\}$, where $|R^{\prime }|=5$, as the nonsequential reference set.*

Therefore, given *R* or $R^{\prime }$, we can train the indoor positioning model $f\left(X^{\left(t\right)}\right)={\hat{s}}^{\left(t\right)}$ for a classification with the target semantic position $s^{\left(t\right)}$ and input RSSI vectors $X^{\left(t\right)}$. Hence, we can predict a sequence of semantic positions $\hat{s}$ from an unlabeled sequence of RSSI vectors $T=<\left(t_{1},X^{\left(1\right)}\right),\left(t_{2},X^{\left(2\right)}\right),...,\left(t_{\left|T\right|},X^{\left(|T|\right)}\right)>$.

### 3.3. Indoor Semantic Trajectory

Given an indoor environment, simply represented by an indoor map, a user manually segments the whole indoor area into a set of nonoverlapping areas $S=\{s_{1},s_{2},s_{3},...,s_{N}\}$ in terms of contextual and geographical information.

An indoor floor map contains contextual and geographical information of the indoor space. The geographical information of the indoor space includes several features such as building and room shapes, positions, surroundings, and obstructing objects (walls or any separator). Thus, we can extract the possibility (or impossibility) to reach a place from a nearby place directly. In contrast, the context gives us the semantic meaning of the areas in the indoor space, such as toilet, corridor, resting area, and exhibition area. If we combine these information, we can define the desired nonoverlapping areas in the indoor space. Each nonoverlapping area denotes a *semantic position*.

A person who is aware of the indoor space and its details, such as a museum manager, can define the semantic positions. In future usage, we can analyze important patterns that represent visitor behavior in the indoor space from the visited semantic positions.

With this notation, we define a semantic trajectory and movement as follows.

**Example** **4.** *According to the contextual and geographical meaning (area names) of the floor map in Figure 1, there are seven different semantic positions. Thus, S={ Room A-North, Room A-South, Corridor AB, Room B-North, Room B-South, Corridor BD, Room D }.*

*We separate Rooms A and B into northern and southern parts because each room has a large area to cover even though they are not obstructed by any object or wall. The separation is actually useful for Room B because it is impossible to reach corridor BD from northern part of Room B directly.*

**Definition** **6.** *The semantic trajectory ST of a moving user is a sequence of timestamped semantic positions $\langle \left(t_{1},s^{\left(1\right)}\right),\left(t_{2},s^{\left(2\right)}\right),...,\left(t_{T},s^{\left(|ST|\right)}\right)\rangle$, where $t_{i}<t_{j}$ when i<j and an element $\left(t_{i},s^{\left(i\right)}\right)$ describes a user to be at a semantic position $s^{\left(i\right)}\in S$ at time $t_{i}$.*

**Definition** **7.** *Given a semantic trajectory ST, we define a movement $s^{\left(i\right)}\to s^{\left(i+1\right)}$, where 1≤i<|ST|, as a displacement of a user from a semantic position $s^{\left(i\right)}$ to $s^{\left(i+1\right)}$ in a consecutive timestamp $t_{i},t_{i+1}$.*

**Example** **5.** *A visitor in a museum walks in a similar fashion-like ground truth in Figure 1b. Thus, the visitor has a semantic trajectory ST=$\langle \left(t_{1},CorridorAB\right),\left(t_{2},RoomB-South\right),\left(t_{3},RoomB-North\right)\rangle$. We can also extract his/her movements from the semantic trajectory ST, which are CorridorAB→B−South and RoomB−South→RoomB−North.*

Then, we introduce the concept of movement constraints by considering the set of neighboring semantic positiosn $NS(s_{i})$ of a semantic position $s_{i}\in S$, the set of movement constraints $E(s_{i})$, and the semantic graph SG.

**Definition** **8.** *We can define a neighbor of a semantic position $s_{i}$ if and only if there is a pair of semantic positions $\langle s_{i},s_{j}\rangle \in S$, where a semantic position $s_{j}$ is adjacent to $s_{i}$ and not obstructed by objects such as walls or furnitures. The neighboring semantic positions have the property of symmetry. Note that i and j are not necessarily inequal.*

**Definition** **9.** *Given a semantic position $s_{i}\in S$, the set of neighboring semantic positions of $s_{i}$, defined as $NS(s_{i})$, is a set of semantic positions where all semantic positions $s_{j}\in NS\left(s_{i}\right)\wedge s_{j}\in S$ and the pair $\langle s_{i},s_{j}\rangle$ hold the relationship of neighboring semantic positions.*

**Definition** **10.** *Given a semantic position $s_{i}\in S$ and its neighboring set $NS(s_{i})$, the set of movement constraints of $s_{i}$, defined as $E\left(s_{i}\right)=\left\{s_{i}\to s_{j},s_{j}\in NS\left(s_{i}\right)\right\}$, consists of all possible movements from $s_{i}$ to $NS(s_{i})$. The movement constraint for the reverse direction $s_{j}\to s_{i}$ also holds true as $NS(s_{j})$ always includes $s_{i}$ (symmetric).*

**Example** **6.** *In Figure 1, the semantic position Corridor AB has a set of neighboring semantic positions NS(CorridorAB)={RoomB−South,RoomB−North,CorridorAB,RoomA−North,RoomA−South}.*

*The indoor positioning technique without constraint infers CorridorBD as the next position at $t_{2}$ from the position CorridorAB at $t_{1}$. However, to access the semantic position Corridor BD from Corridor AB, a user must visit Room B-South first. Thus, Corridor BD is not in the set of neighboring semantic positions of Corridor AB and the movement CorridorAB→CorridorBD violates the movement constraint of Corridor AB, which was formally defined as CorridorBD∉NS(CorridorAB) and CorridorAB→CorridorBD∉E(CorridorAB), respectively.*

**Definition** **11.** *A semantic graph SG consists of a tuple (S,E), where S is a set of semantic positions and E is a set of movement constraints of all semantic positions in S. A semantic position $s_{i}\in S$ and a set of movement constraints $E(s_{i})\in E$ in semantic graph represent a vertex and the undirected edges from the respective vertex in a graph, respectively. Although they have directional properties, the movement constraints are simplified as undirected edges as they hold the symmetric relationship $s_{i}\to s_{j}$ and $s_{j}\to s_{i}$.*

**Figure 4:**
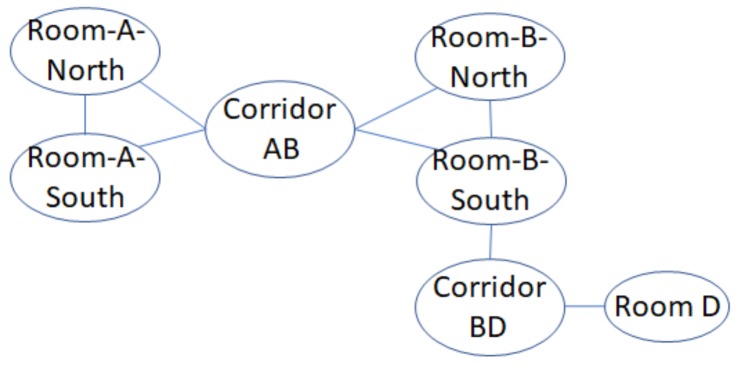
Semantic graph of the floor plan in Figure 1a.

**Example** **7.** *Semantic graph representation of the floormap in Figure 1 is depicted in Figure 4. We draw each semantic position in S as vertices. Then, for each vertex s∈S, we establish movement constraints E(s) to its neighboring semantic position set NS(s) as undirected edges. If a movement constraint already exists from $s_{i}$ to $s_{j}$ ($s_{i},s_{j}\in S$), the movement constraint from $s_{j}$ to $s_{i}$ should not be drawn.*

Then, we define the invalid trajectory and, consequently, the valid trajectory.

**Definition** **12.** *A semantic trajectory ST is an invalid trajectory that contains at least a movement $\exists s^{\left(i\right)}\to s^{\left(i+1\right)},1\leq i<\left|ST\right|$ that violates the movement constraint of $s^{\left(i\right)}$ in semantic graph SG(S,E) such that $s^{\left(i\right)}\to s^{\left(i+1\right)}\notin E\left(s^{\left(i\right)}\right),s^{\left(i\right)}\in S$ exists.*

**Definition** **13.** *A semantic trajectory ST is a valid trajectory when all of its movements $\forall s^{\left(i\right)}\;\to \;s^{\left(i+1\right)}$, 1≤i<|ST| always complies in any movement constraint of $s^{\left(i\right)}$ in the semantic graph SG(S,E) such that $s^{\left(i\right)}\to s^{\left(i+1\right)}\in E\left(s^{\left(i\right)}\right),s^{\left(i\right)}\in S$.*

**Example** **8.** *In Figure 1c, we see an actual semantic trajectory and an estimated semantic trajectory from an indoor positioning technique (without constraint). The actual semantic trajectory is a valid trajectory as all of its movements { Corridor AB → Room B-South, Room B-South→ Room B-North} do not violate any movement constraint of {E(CorridorAB),E(RoomB−South)} respectively. The estimated semantic trajectory is an invalid trajectory as its two movements {Corridor AB → Corridor BD, Corridor BD → Room B-North} violate the movement constraints of {E(CorridorAB),E(CorridorBD)} respectively.*

Then, we continue to define the semantic trajectory extraction using the previously mentioned notions.

**Definition** **14.** *Semantic trajectory extraction. Given a timestamped sequence of RSSI vector $T=<\left(t_{1},X^{\left(1\right)}\right),\left(t_{2},X^{\left(2\right)}\right),...,\left(t_{\left|T\right|},X^{\left(|T|\right)}\right)>$ and a trained indoor positioning model f with a reference set R or $R^{\prime }$ as training set, we estimate a semantic trajectory $\hat{ST}=$$<(t_{1},{\hat{s}}^{\left(1\right)}),$$\left(t_{2},{\hat{s}}^{\left(2\right)}\right),...,\left(t_{\left|T\right|},{\hat{s}}^{\left(|ST|\right)}\right)>$ where each predicted semantic position ${\hat{s}}^{\left(t\right)}\in S$ corresponds to the indoor positioning function $f\left(X^{\left(t\right)}\right)={\hat{s}}^{\left(t\right)},1\leq t\leq \left|T\right|$ and |T|=|ST|.*

## 4. Proposed Method

In this section, we describe the details of our approach for extracting semantic trajectories from the deployed RSSI beacons.

### 4.1. Architectural Overview

**Figure 5:**
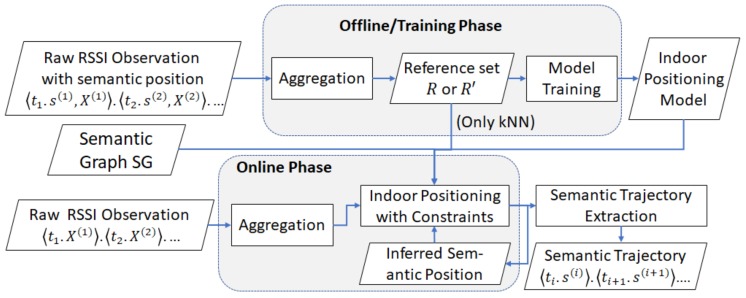
System architecture of our approach.

We show the architectural overview of our constrained approach in Figure 5. The main inputs of our system are a semantic graph and the raw RSSI observations. The semantic graph is manually defined by users and the raw RSSI observations are acquired from a smartphone, which acts as RSSI readers. In the system, the semantic graph is represented by an adjacency matrix.

The two phases refer to (1) the offline phase, which contains the reference set collection and indoor positioning model training, and (2) the online phase, i.e., real-time indoor semantic trajectory extraction.

### 4.2. Data Collection

The BLE RSSI data collection is handled by a person manually. We deploy *M* BLE beacons across the indoor environment. The collector captures BLE RSSI vectors by a smartphone. Then, the collector walks from an initial point (the most possible entrance, for example, stairs/elevator/main door) and goes to his/her destination inside the building. There are different movements based on the users’ role and characteristics. Some of them are as follows: (1) the visitors in a museum may see all the exhibition objects, (2) some visitors may not have the time to explore all the objects and may exit the museum earlier, and (3) some staff may go back and forth, checking every object’s condition. The collector already knows the semantic graph of the studied environment. Thus, while the collector is walking around the environment, he/she labels his/her current semantic position using the smartphone. If the collector moves to another position, then he/she changes the current semantic position. Hence, we acquire trajectory data in the form of tuples of the timestamp, the raw BLE RSSI observation from the beacons, and the semantic position.

We apply an aggregation technique to both stages as a preprocessing technique. The aggregation improves the quality of the RSSI readings with less missing observations and more stable RSSI values.

### 4.3. Aggregation

The observation of our use case uses BLE RSSI, which is often missing and varies in an indoor space with many surrounding objects. Thus, to overcome these issues, we employ a sliding aggregation window to gain statistical information for consecutive signal strength samples. Aggregation functions such as mean or max function can provide such statistical information.

An aggregation window with length *l* performs an aggregation function on a set of raw observations $X^{\left({\bar{t}}_{j}\right)}$. The set $X^{\left({\bar{t}}_{j}\right)}$ contains the observations from one or more timestamps that span from $t_{i-a}$ to $t_{i}$, given an integer a≥0 that maximizes $t_{i-a}-t_{i}$ and satisfies $t_{i-a}-t_{i}\leq l$. Note that ${\bar{t}}_{j}=t_{i}$ is the latest timestamp in the set. Then, the aggregation function on the set $X^{\left(\bar{t}\right)}$ produces a pair $\langle {\bar{t}}_{j},{\bar{X}}^{\left(j\right)}\rangle$.

To produce the next aggregated pair $\langle {\bar{t}}_{j},{\bar{X}}^{\left(j\right)}\rangle$, we shift the aggregation window to $l^{+}$ seconds forward. Thus, we define ${\bar{t}}_{j+1}$ equal to $t_{i+b}$, given an integer b≥1 that minimizes $t_{i+b}-t_{i}$ and satisfies $t_{i+b}-t_{i}\geq l^{+}$. Hence, we can perform the next aggregation on the set of raw observations $X^{{\bar{t}}_{j+1}}$. We apply these steps to the set of raw BLE observations.

**Figure 6:**
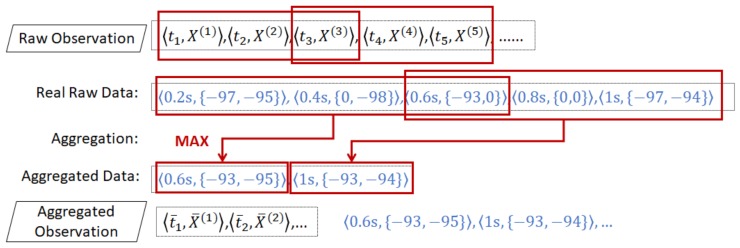
Performing aggregation from raw observations.

**Example** **9.** *Figure 6 illustrates an example of aggregation using 2 BLE beacons in a one-second sampling. We see five observations from time 0.2 s to 1 s. At each timestamp, we have an RSSI value from two beacons in the format $\langle t_{i},\left\{x_{1}^{\left(i\right)},x_{2}^{\left(i\right)}\right\}\rangle$. The first observation $\langle t_{1},X^{\left(1\right)}\rangle$ is 〈0.2s,{−97,−95}〉.*

*We assume that the length of the aggregation window is 0.6 s and that the sliding interval is 0.4 s. We start the first aggregation on the third observation to the beginning, where the aggregation windows spans from 0 s to 0.6 s $\left(t_{3}-t_{1}\leq 0.6\;s,a\;=2\right)$. Then, we slide the aggregation window 0.4 s forward to 1 s $\left(t_{5}-t_{3}\geq 0.4\;s,b=2\right)$. Thus, the second aggregation is from the fifth observation to the third observation, from 0.6 s to 1 s.*

*For each aggregation window, we perform the max aggregation function on all RSSI values of each beacon. The first aggregation on the first beacon RSSI value yields −93 from three values: {−97,0,−93}. We exclude the missing value (0) from the aggregation function. Similarly, for the second beacon, we get −95 from {(−95,−98,0}. Then, we get 〈0.6s,{−93,−95}〉 as the first aggregated observation $\langle {\bar{t}}_{1},{\bar{X}}^{\left(1\right)}\rangle$. Using the same procedure, we get $\langle {\bar{t}}_{2},{\bar{X}}^{\left(2\right)}\rangle =\langle 1s,\left\{-93,-94\right\}\rangle$ as the second aggregated observation.*

However, missing observations from all installed beacons ($X^{\left(t\right)=\left\{{\bar{x}}_{i}^{\left(t\right)}|{\bar{x}}_{i}^{\left(t\right)}=0\wedge 1\leq i\leq M\right\}}$) can occur even though we performed the aggregation. In this case, it is still possible to infer the position based on the prediction of indoor positioning techniques. We have trained the indoor positioning techniques to learn this problem because the training/reference set may contain some missing observations. The constraints also hold a sequential property to prevent impossible transitions due to the all-missing observations.

### 4.4. Offline Phase

In the offline phase, we collect the reference set $R=\{T_{1},$$T_{2},...,T_{\left|R\right|}\}$, where $T_{i}=<\left(t_{1},X^{\left(1\right)},s^{\left(1\right)}\right),$$\left(t_{2},X^{\left(2\right),s^{\left(2\right)}}\right),...,\left(t_{T_{i}},X^{\left(|T_{i}|\right)},s^{\left(|T_{i}|\right)}\right)>,T_{i}\in R$, from the raw observations and known semantic positions. We preprocess the raw RSSI observation of the reference set using the aggregation window. We also aggregate the semantic positions. The aggregation of the semantic positions is slightly different from the aggregation of the raw observations.

To aggregate the semantic positions, we perform the majority vote on $S^{{\bar{t}}_{j}}$, the set of semantic positions covered by an aggregation window at time ${\bar{t}}_{j}$. We denote this aggregated reference position by ${\bar{s}}^{\left(j\right)}$. If a tie occurs between some semantic positions, we randomly pick any of the top majority candidates as an aggregated label. Thus, we can define each labeled observation as a triplet $\left({\bar{t}}_{j},{\bar{X}}^{\left(j\right)},{\bar{s}}^{\left(j\right)}\right)$, maintaining the relationship similar to the raw observations.

With this reference set *R*, or simply transformed to $R^{\prime }$, we can perform the semantic position estimation directly (using kNN) or train the indoor positioning models first (for the approaches other than kNN) using our constrained indoor positioning approach in the online phase.

### 4.5. Online Phase

In the online phase, we perform semantic trajectory extraction on the captured RSSI observation using movement constraints. In the real world, we show this extracted semantic trajectory on the user’s mobile device while the user is still inside the building. Thus, it is essential to have an efficient real-time processing technique for semantic trajectory extraction. In this approach, we apply the constraints to three different styles of semantic indoor positioning models: HMM, kNN, and the other approach. We guarantee these approaches to work in real-time.

#### 4.5.1. HMM Using Online Viterbi with Constraints

The Hidden Markov Model (HMM) with the Viterbi algorithm can decode a hidden sequence from a sequence of observations. In this case, we use multivariate HMM (MHMM) to estimate the most likely semantic trajectory $\hat{ST}$ from a sequence of timestamped RSSI vectors $T=<({\bar{t}}_{t},{\bar{X}}^{\left(t\right)})|1\leq t\leq |T|>$ as the input. We use MHMM because we deploy more than one beacon; thus, we need to observe multiple devices. Then, we train the MHMM model using the reference set *R* (as set of trajectories). We summarize the description of the components of HMM in Table 1.

We describe the relationship between the MHMM transition probability matrix *A* and movement constraints in semantic graph SG in Equation (1). If a movement from a semantic position $s_{i}$ to $s_{j}$, where $s_{i},s_{j}\in S$ is not possible, violates the movement constraint, the transition probability of $s_{i}$ to $s_{j}$ is zero.

(1) $$A\left(i,j\right)=0\;\mathrm{when}\;s_{i}\to s_{j}\notin E\left(s_{i}\right)\wedge s_{i},s_{j}\in S$$

In contrast, we set the size of the emission probability matrix to |S|×M×K, where *M* is the number of deployed beacons and *K* is the possible emitted RSSI within the range of negative integers and 0. We limit these values from −1 to −100, where any value lower than −100 is categorized as 0. Thus, we fix the value of K=101, where the additional value stands for the missing value (0).

Originally, an HMM uses the Viterbi algorithm to see the full trajectory to infer the best solution from the sequence of observations. However, the traditional Viterbi algorithm cannot immediately output an optimal solution in real time because the full trajectory can only be seen after the user finishes his/her trip inside the building. Thus, we apply an online Viterbi approach similar to Reference [11] with modifications to extract the most likely subsequence given a window with length *w*.
**Algorithm 1: **Initialization algorithm of Hidden Markov Model (HMM) with constraint.
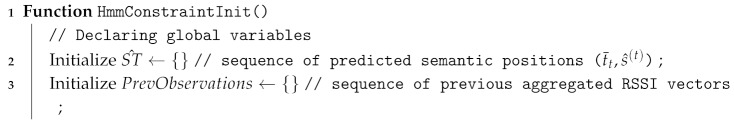


Before we perform the online Viterbi of the HMM with constraint, we initialize the global variables of the HMM with constraint in Algorithm 1. The global variables store the information of the previously seen observations (PrevObservations) and predicted semantic positions ($\hat{ST}$). Then, we perform the online Viterbi of HMM with constraint in Algorithm 2 whenever an RSSI vector is captured.

We describe the online viterbi of HMM with constraint (Algorithm 2) as follows. The original Viterbi is performed on the subset of the observation sequences with length *w* from the observation at time t−w+1 to current observation at time *t* inclusively. Consequently, we cannot perform the online Viterbi (lines 3–4) unless the timestamp of the current observation is the (w+1)-th observation (line 5). Thus, defining *w* is important as a longer *w* should have closer optimality to the full trajectory but a longer *w* also makes the prediction slower and delayed. After that, we subset the previous observation according to the condition if the current observation is at the end of trajectory (lines 6–11). Lined 12–14 represents the application of the constraint to the HMM model. For the beginning of the trajectory (predicting t=1), we should use π for initial probability as default. However, in the later part, we already predicted our previous position as ${\hat{s}}^{\left(t-1\right)}$. Hence, we set the π as the transition probability of ${\hat{s}}^{\left(t-1\right)}$ for the Viterbi in the later part of the trajectory (line 14). Then, we compute the suboptimal solution using the Viterbi at line 16. If the trajectory does not end, we add the predicted positions one by one because we might see some change in the solution when the next observation ${\bar{X}}^{\left(t+1\right)}$ comes (lines 17–18). If the trajectory ends, we directly add all of the predicted positions to the predicted semantic trajectory $\bar{ST}$.
**Algorithm 2: **Algorithm of online Viterbi in HMM with constraint
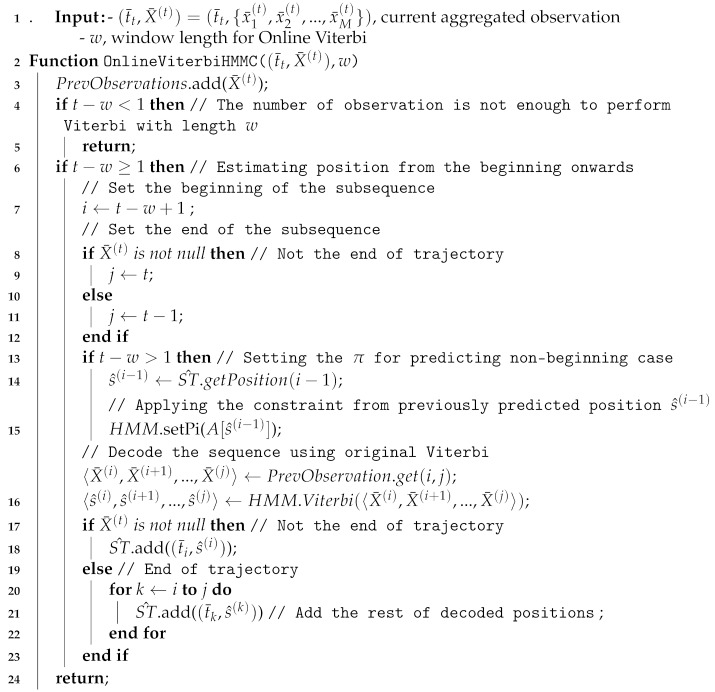


Thus, we obtain the predicted semantic trajectory $\bar{ST}$ as the concatenated output of the online Viterbi.

#### 4.5.2. k-Nearest Neighbor with Movement Constraints

The k-Nearest Neighbor with movement constraints (kNN-C) estimates a semantic position ${\hat{s}}^{\left(t\right)}$ from the streamed aggregated observation $\langle {\bar{t}}_{t},{\bar{X}}^{\left(t\right)}\rangle$ at timestamp ${\bar{t}}_{t}$ from the reference set $R^{\prime }$, a semantic graph $SG\left(S,E\right)$, and the previously estimated semantic position ${\hat{s}}^{\left(t-1\right)}$. Given the previously estimated semantic position ${\hat{s}}^{\left(t-1\right)}$, we can remove irrelevant observations from the reference set $R^{\prime }$. Thus, we can output the close semantic position, thereby preventing the occurence of invalid trajectories.

The main difference between kNN with and without the movement constraints is the search space. The kNN without constraints considers all observations in the reference set as the search space whereas that with constraints only checks the reference set that consists of the members of the neighboring set of semantic positions $NS({\hat{s}}^{\left(t-1\right)})$ (the previously inferred position ${\hat{s}}^{\left(t-1\right)}$). Therefore, applying constraints to kNN ensures search space reduction and validity of semantic trajectory.

**Figure 7:**
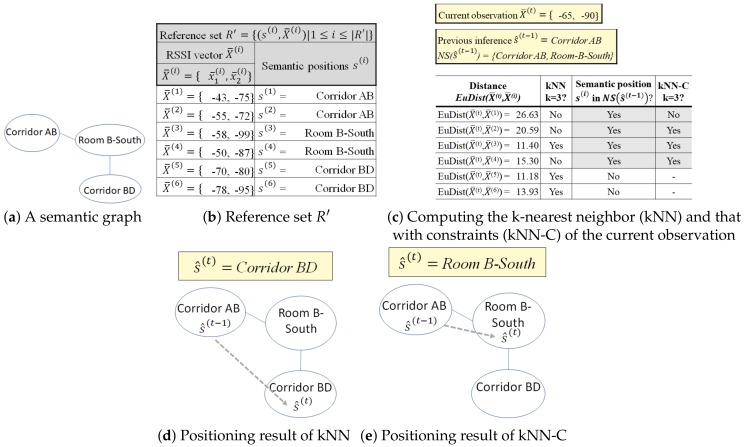
Effects of movement constraints on kNN.

**Example** **10.** *In Figure 7, we apply both kNN and kNN-C with k=3 for an indoor environment with two installed RSSI beacons (M=2). We consider a subset of the semantic graph from Figure 4, depicted in Figure 7a. We describe the reference set $R^{\prime }$ in Figure 7b. From data collection, we have two aggregated observations at each semantic position in the reference set $R^{\prime }$; thus, $|R^{\prime }|=6$.*

*Figure 7c depicts that the current aggregated RSSI vector is ${\bar{X}}^{\left(t\right)}$, the previously inferred position ${\hat{s}}^{\left(t-1\right)}$, and the distance computation for kNN-based methods. The current observation ${\bar{X}}^{\left(t\right)}$ contains two RSSI values {−65,−90} from two beacons. The previously inferred semantic position ${\hat{s}}^{\left(t-1\right)}$ is at Corridor AB; thus, its neighboring semantic areas $NS\left(CorridorAB\right)$ are {RoomB−South,CorridorAB}. Then, we compute the distances between the current observation ${\bar{X}}^{\left(t\right)}$ with the instances ${\bar{X}}^{\left(i\right)}$ in $R^{\prime }=\left\{RoomB-South,CorridorAB\right\}$ as the search space. Note that $EuDist({\bar{X}}^{\left(t\right)},{\bar{X}}^{\left(i\right)})$ measures the Euclidean distance of the RSSI vectors between current observation ${\bar{X}}^{\left(t\right)}$ and the instances of ${\bar{X}}^{\left(i\right)}$ in reference set $R^{\prime }=\{\left(s^{\left(i\right)},{\bar{X}}^{\left(i\right)}\right)\left|1\leq i\leq \right|R^{\prime }\left|\right\}$ and may not reflect the actual geographical distance. When we use kNN without the movement constraint, we compare ${\bar{X}}^{\left(t\right)}$ to six observations in $R^{\prime }$ in the reference set. However, by applying the movement constraint to the kNN, we have to check only four references from $NS\left(CorridorAB\right)$ (shaded by light gray in Figure 7c). By doing this, we reduce the search space for the comparison and ensure the validity of the extracted trajectory.*

*The kNN (k=3) takes three members from reference set $R^{\prime }$ with the smallest distance from the current measurement ${\bar{X}}^{\left(t\right)}$. Thus, it obtains two instances with the semantic position Corridor BD and one instance with the semantic position Room B-South as the nearest neighbors. Meanwhile, kNN-C only checks the references in $R^{\prime }$ that are included in $NS\left(CorridorAB\right)$; thus, it only considers two instances with the semantic position Room B-South and one instance with the semantic position Corridor AB as the nearest neighbors. Using a majority vote, kNN returns Corridor BD while kNN-C returns Room B-South as the estimated semantic position. The extracted movement of kNN, as depicted in Figure 7d, RoomB−North→CorridorBD violates the movement constraint from Room B-North whereas the extracted movement of kNN-C, RoomB−North→RoomB−South, depicted in Figure 7e, does not. Thus, kNN-C provides a valid trajectory.*

Algorithm 3 describes the application of movement constraints to the kNN-based method. The algorithm reduces the search space in $R^{\prime }$ by the movement constraints using the previously inferred semantic position ${\hat{s}}^{\left(t-1\right)}$ as input in line 1. We perform the kNN method on the subset of $R^{\prime }$, which contains only $NS({\hat{s}}^{\left(t-1\right)})$, i.e., the constrained reference set $R_{c}^{\prime }$ in line 8. The set $R_{c}^{\prime }$ represents the reduced search space when ${\hat{s}}^{\left(t-1\right)}$ is available. Then, we perform the naïve kNN on set $R_{c}^{\prime }$ (line 11). If ${\hat{s}}^{\left(t-1\right)}$ is not available $\left(t=1\right)$, which represents the beginning of the trajectory, we cannot reduce the search space and perform naïve kNN using the original *R* (line 5).
**Algorithm 3: **Applying a constraint to kNN.
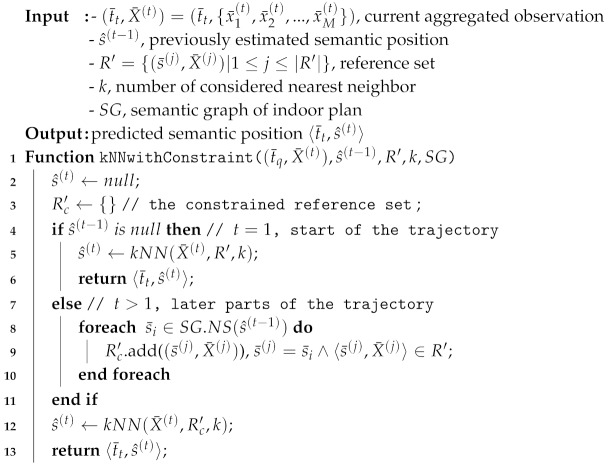


#### 4.5.3. Other Indoor Positioning with Constraints

We assume an indoor positioning task as a multi-class prediction of a machine learning model. In this case, we discuss the general machine learning model besides HMM and kNN. The models can vary from Deep Neural Network (DNN), Support Vector Machine (SVM), Logistic Regression, and others as long as they can perform classification tasks. We train an indoor positioning model by $R^{\prime }$ to produce a likelihood model. The model predicts a semantic position ${\hat{s}}^{\left(t\right)}\in S$ given an input of aggregated RSSI vector ${\bar{X}}^{\left(t\right)}$ at time *t*. The output ${\hat{s}}^{\left(t\right)}$ is a semantic position *s* where $s=\arg\;\max_{s\in S}P\left(s|{\bar{X}}^{\left(t\right)}\right)$ and $\sum_{s\in S}P\left(s|{\bar{X}}^{\left(t\right)}\right)=1$. $P(s|{\bar{X}}^{\left(t\right)})$ is the likelihood of a semantic position *s* given the input ${\bar{X}}^{\left(t\right)}$ at time *t*, which is the result of training the indoor positioning model.

Then, given the result of the trained indoor positioning model, we can ensure the validity of the extracted trajectory using the indoor positioning and adding movement constraints to the previously predicted semantic position ${\hat{s}}^{\left(t-1\right)}$. We add ${\hat{s}}^{\left(t-1\right)}$ as another input to the indoor positioning; thus, we get $f\left(X^{\left(t\right)},{\hat{s}}^{\left(t-1\right)}\right)={\hat{s}}^{\left(t\right)}$ as the indoor positioning formula. Then, we formulate the output, i.e., current predicted semantic position ${\hat{s}}^{\left(t\right)}$, in Equation (2).

(2) $$f\left(X^{\left(t\right)},{\hat{s}}^{\left(t-1\right)}\right)={\hat{s}}^{\left(t\right)}=\arg\;\max_{s\in S}\left\{\begin{array}{c} P\left(s|{\bar{X}}^{\left(t\right)}\right),{\hat{s}}^{\left(t-1\right)}\to s\in E\left({\hat{s}}^{\left(t-1\right)}\right)\\ 0,\mathrm{otherwise} \end{array}\right.$$

We only apply the likelihood of the semantic positions *s* when ${\hat{s}}^{\left(t-1\right)}\to s\in E\left({\hat{s}}^{\left(t-1\right)}\right)$. Thus, we guarantee the extraction of the valid trajectory as output using the movement constraint.

We provide the algorithm for the constrained ML approach in Algorithm 4.
**Algorithm 4: **Applying a constraint to a machine learning (ML) classification task.
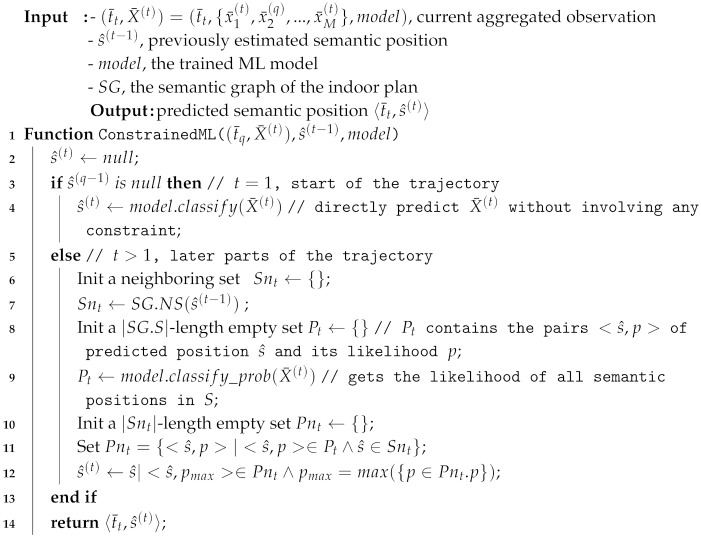


Note that, similar to the constrained kNN, when t=1 in the beginning, we only perform a prediction without ${\hat{s}}^{\left(t-1\right)}$ and the constraints.

### 4.6. Semantic Trajectory Extraction

Finally, we have the result of indoor positioning, i.e., the current estimated semantic position $\langle {\bar{t}}_{t},{\hat{s}}^{\left(t\right)}\rangle$. Then, we continuously concatenate $\langle {\bar{t}}_{t},{\hat{s}}^{\left(t\right)}\rangle$ from the beginning until the end of trajectory *T* as the predicted trajectory $\hat{ST}$. We denote the inferred semantic trajectory by $\hat{ST}=$$<(t_{1},{\hat{s}}^{\left(1\right)}),$$\left(t_{2},{\hat{s}}^{\left(2\right)}\right),...,\left(t_{|\hat{ST}|},{\hat{s}}^{\left(|\hat{ST}|\right)}\right)>$. Note that all of the studied indoor positioning techniques perform in real time. Hence, the user can see his/her visited semantic positions while he/she is still walking inside the building.

## 5. Experimental Results

In this section, we present the setting of our experiments and the performance evaluation of our approaches. Our goal is to evaluate the efficiency and effectiveness of the constrained approach for extracting valid semantic trajectories from the installed BLE beacons in an indoor environment. In this experiment, we use Deep Neural Network (DNN) for the base of the other ML-based indoor positioning technique. We compare the performance of our approach of HMM with online Viterbi and constraints (Section 4.5.1), kNN with constraints (Section 4.5.2), and DNN with constraints (Section 4.5.3) to the baseline naïve kNN method, adaptive bandwidth mean shift + kNN [8], unsupervised multivariate HMM, simple DNN, and particle filter (PF). We choose particle filter as a non-machine learning approach in indoor positioning.

We implement all approaches from scratch using Java except for DNN, which is provided by Tensorflow 1.15. We use a machine equipped with Intel(R) Core i7 3.6 GHz CPU and 16 GB RAM. We summarize the approaches in Table 2. We format our proposed approaches with bold typeface in the table.

### 5.1. Dataset

**Figure 8:**
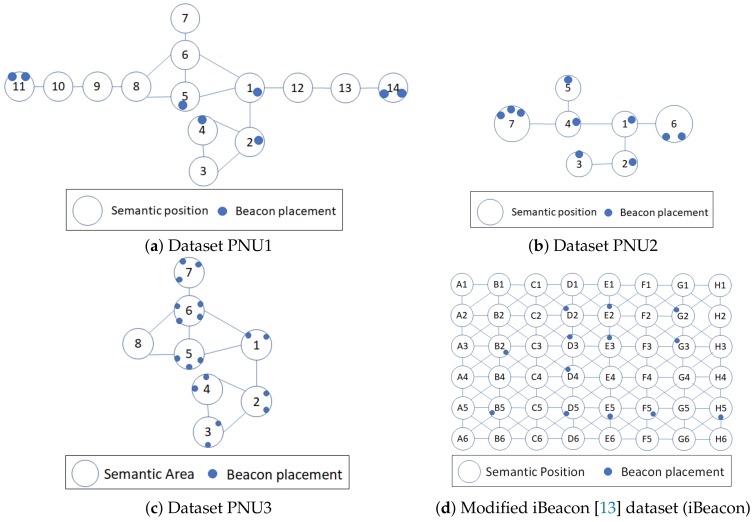
Semantic graphs of the collected datasets

We used real trajectory-BLE RSSI datasets from two different indoor environments: (1) the fourth floor of our campus building and (2) the first floor of a library building [13]. For the first environment, we collected three datasets with different beacon deployments and semantic position definitions. The data collection in the first environment used Beabig BLE beacons with the Bluno firmware v. 1.8 [21] and an Android OS 8.10 smartphone with 3 GB RAM, 1.6 GHz processor, and sampling rate of 200 ms each. The semantic graphs of PNU1, PNU2, and PNU3 are depicted in Figure 8a–c, respectively. The PNU1, PNU2, and PNU3 datasets have different definitions of semantic positions and beacon placements.

For the second environment, we modified the iBeacon dataset (iBeacon) to suit our case because this dataset is originally for stay estimations. We defined the semantic positions in as depicted in Figure 8d. We also defined the neighboring set of each semantic position as the 8-direction connectivity, for example, NS(C3)={B2,B3,B4,C2,C3,C4,D2,D3,D4}. We extracted trajectories from the iBeacon dataset using the time and area threshold as it provides timestamps for each recorded BLE observation. Even though it is provided for stay estimation, it captures some movements that can be used for our problem setting.

A detailed summary of all datasets is given in Table 3. We subset some trajectories as the reference or training set and used the others as the test set. We performed the trajectory extraction on the test set to measure the quality of our approach. Note that the numbers in the reference and the test set represent the number of trajectories, while the total length denotes the total of all semantic positions in all trajectories (reference + test). Note that we use the PNU3 dataset to identify the performance of our approach on a dynamic environment.

The dynamic environment stands for the environment in which the PNU3 dataset consists of three different settings of the number of people walking in the indoor space, which are 5, 10, and 13 people. The dynamic environment experiment is conducted as follows. We use the 10-people dataset as the reference set and training set. Then, we test our trained model to the combination of two different test sets (5 and 13 people test sets). We compare the performance to the original training set for each number of people. Thus, given that setup, we can see the performance of the indoor positioning techniques to dynamic environment.

Before performing the main experiments, we would like to study the effect of the weak RSSI threshold to the indoor positioning techniques. We use kNN and kNN with constraint methods with PNU1 dataset for this preliminary study. Based on the preliminary experiment result, we use the best value of the threshold to perform the main experiments.

### 5.2. Parameter Setup

Our approach requires some parameters for the aggregation window and the indoor positioning method. For brevity, we have not included the study of the aggregation window parameters. Thus, we fix the aggregation parameters: aggregation window length (*l*) = 1 s, sliding length ($l^{+}$) = 0.5 s, and max aggregation function. The max function considers the strongest RSSI value of each beacon in the aggregation window.

We do not use the mean function because a large counterpart of the datasets contains missing observations. Performing mean on a sequence with missing values yields similar statistical information to that of the max function (averaging one or two nonzero values).

We compare each indoor positioning method style with its respective constrained addition (except for HMM). For the HMM, we compare the constrained approach with the basic unsupervised approach, which sees the full trajectory (not suitable for real-time environments). We study the behaviour of HMM-C by varying its window length. We use naïve kNN and the ABMS + kNN [8] as the baseline approaches for kNN because they yield good results in 2D positioning cases. However, these methods do not perform well in the real-time setting. Thus, we also study their efficiency. Additionally, we cannot directly apply our movement constraints technique to ABMS + kNN because ABMS + kNN requires 2D coordinates. Instead, we convert the final result of kNN in ABMS + kNN into the closest semantic position, which has the smallest distance from the semantic position’s centroid to the inferred position.

Table 4 describes the settings of parameters used in the experiment. ABMS + kNN requires additional parameters, i.e., bandwidth and number of groups, which were set as 10 and 20, respectively. The particle filter uses 100 particles, 1.2 m/s walking speed, and a gaussian distribution noise with 0 mean and 0.3 standard deviation. We report the behaviour of the approaches according to the varied parameters.

### 5.3. Evaluation Metrics

To measure the performance of each approach, we used three metrics in terms of effectiveness and validity. We applied these metrics to an extracted semantic trajectory $\hat{ST}$ and its respective ground truth ST, where $ST=\langle (t_{t},s^{\left(t\right)})\rangle$, $\hat{ST}=\langle (t_{t},{\hat{s}}^{\left(t\right)})\rangle$, and 1≤t≤|ST|.

#### 5.3.1. Error

We used a simple classification error to measure the correctness of the predicted semantic trajectory $\hat{ST}$ to the respective ground truth ST. Both had the same length |ST|. The formula of error is given in Equation (3).

The value of Err ranges from 0 to 1, where 0 stands for an entirely correct prediction for a single trajectory whereas 1 is obtained if the positioning method mispredicts all semantic positions to be different semantic positions.

(3) $$Err\left(\hat{ST},ST\right)=\frac{1}{\left|ST\right|}\sum_{t=1}^{\left|ST\right|}\left\{\begin{array}{c} 1,{\hat{s}}^{\left(t\right)}\neq s^{\left(t\right)}\\ 0,{\hat{s}}^{\left(t\right)}=s^{\left(t\right)} \end{array}\right.$$

Note that we are not only interested in correctly predicted positions as defined by the smallest error possible. Instead, we are intrigued by applying the commonly used positioning error in 2D indoor positioning for semantic positions. The positioning error should measure how far apart the predicted position is from the ground truth. In the semantic position case, this relationship is reflected by the semantic distance $d_{SG}\left({\hat{s}}^{\left(t\right)},s^{\left(t\right)}\right)$. Thus, we think that only the error is not sufficient in capturing the effectiveness of the semantic positioning. Hence, we define another metric called Semantic Positioning Error Rate (SPER).

#### 5.3.2. Semantic Positioning Error Rate

First, we define the semantic distance $d_{SG}\left(s_{i},s_{j}\right)$ between two semantic positions $s_{i},s_{j}\in S$ as the number of minimum traversed edges from $s_{i}$ to $s_{j}$ in the semantic graph SG. We can compute the distance using the Dijkstra algorithm, given $s_{i}$ as the source and $s_{j}$ as the destination. Thus, we can state that, at time $t_{t}$, an inferred semantic position ${\hat{s}}^{\left(t\right)}$ is correct if its semantic distance to the ground truth $s^{\left(t\right)}$ is zero $\left(d_{SG}\left({\hat{s}}^{\left(t\right)},s^{\left(t\right)}\right)=0\right)$. When $d_{SG}\left({\hat{s}}^{\left(t\right)},s^{\left(t\right)}\right)$ becomes larger than 0, the predicted semantic position gets farther from the ground truth.

As a measure of effectiveness, we compute the relative ratio of the semantic distance of the predicted semantic position ${\hat{s}}^{\left(t\right)}$ to the maximum possible distance/misprediction, given the ground truth $s^{\left(t\right)}$. We call this metric the semantic positioning error rate, $SPER\left({\hat{s}}^{\left(t\right)},s^{\left(t\right)}\right)$, which is described in Equation (4). The value of SPER ranges from 0 to 1, where 0 stands for an entirely correct prediction for a single trajectory whereas 1 is obtained if the positioning method mispredicts all semantic positions as the farthest possible semantic positions.

(4) $$SPER\left(ST,\hat{ST}\right)=\frac{1}{\left|ST\right|}\sum_{t=1}^{\left|ST\right|}\frac{d_{SG}\left(s^{\left(t\right)},{\hat{s}}^{\left(t\right)}\right)}{\max_{1\leq i\leq |S|}d_{SG}\left(s^{\left(t\right)},s_{i}\right)}$$

#### 5.3.3. Validity

The validity measures the “jumpy”-ness of the extracted trajectory. We do not use ground truth ST as it is not related to the distant movement of the extracted trajectory. Similar to SPER, if the consecutive predicted semantic positions are too far, that trajectory is considered to be less valid. We define this measure as $V(\hat{ST})$ in Equation (5).

(5) $$V\left(\hat{ST}\right)=\frac{1}{|ST|-1}\sum_{q=2}^{\left|ST\right|}\left\{\begin{array}{c} 1,{\hat{s}}^{\left(q\right)}\in NS\left({\hat{s}}^{\left(q-1\right)}\right)\\ 1-\frac{d_{SG}\left({\hat{s}}^{\left(q-1\right)},{\hat{s}}^{\left(q\right)}\right)}{\max_{1\leq i\leq |S|}d_{SG}\left({\hat{s}}^{\left(q-1\right)},s_{i}\right)},\;otherwise \end{array}\right.$$

The value of $V(\hat{ST})$ ranges from 0 to 1, where 1 stands for a valid trajectory and 0 is obtained if all of the consecutive semantic positions are the pairs of the farthest possible semantic positions.

### 5.4. Results

First, we discuss the effect of the threshold to an indoor positioning technique performance.

Then, we observe the performance of each indoor positioning with varied parameters on SPER. Note that we also compare the efficiency of the kNN-based methods by search space reduction and the computation time in the online phase. The search space reduction does not affect HMM and DNN methods, unlike kNN, because they estimate the semantic positions using the trained model rather than looking at the reference set. Thus, their computation time would not change significantly. Then, we discuss the difference between the error and SPER of different datasets using each method. Next, we examine the validity of the trajectory extracted via each method. After that, we examine the performance of the indoor positioning techniques with constraint in a dynamic environment.

#### 5.4.1. RSSI Value Thresholding

**Figure 9:**
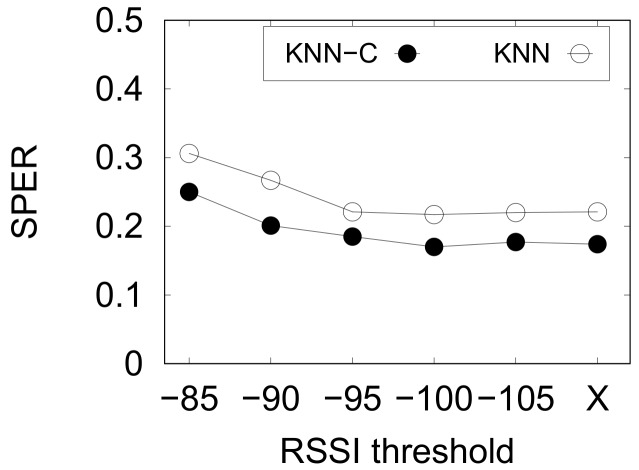
Preliminary study on RSSI value thresholding.

Previously, in Section 3.2, we mentioned that weak RSSI may happen due to several circumstances. However, this might be useful for the ML-based indoor positioning to learn the weak RSSI characteristics. We present the result of this preliminary study using kNN-based methods and PNU1 dataset in Figure 9. We denote no threshold as “X” in the chart.

From Figure 9, it is clear that the threshold value of −100 gives the optimal thresholding value. In contrast, larger thresholds (≥−95) did not improve the performance of indoor positioning because they ignore the weak values of RSSI. Similarly, weaker values or no threshold only give slight enhancement to the performance. Hence, we use the threshold value of −100 in the next experiments.

#### 5.4.2. HMM

We compare the effectiveness of HMM with online Viterbi and constraints (HMM-C) to unsupervised MHMM (HMM) to determine the consistency between the suboptimal results yielded by the online approach with constraint and the optimal results by the original MHMM.

**Figure 10:**
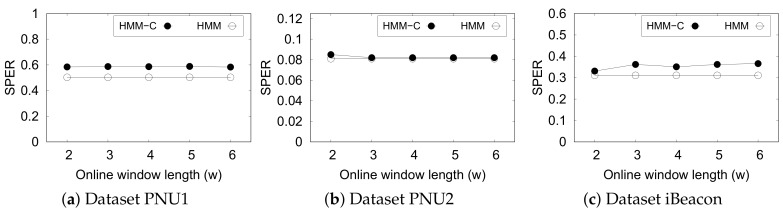
Performance of Hidden Markov Model (HMM) and that with constraints (HMM-C) with various window length.

Figure 10 shows the performance of HMM-based methods based on SPER. Note that the original approach gives straight lines as we perform the estimation using the full trajectory. It is evident that the online MHMM does not exhibit any significant difference if the subsequence window length *w* is increased. However, we do not want to set this parameter at a large value as it delays the prediction time. Overall, HMM-C is comparable to its optimal original approach except in dataset PNU1. This indicates that HMM-C is almost as effective as the original HMM even though it does not see the full trajectory.

#### 5.4.3. kNN

We compare the performance of the kNN-based indoor positioning techniques in terms of effectiveness (SPER) and efficiency (search space reduction and average computation time).

**Figure 11:**
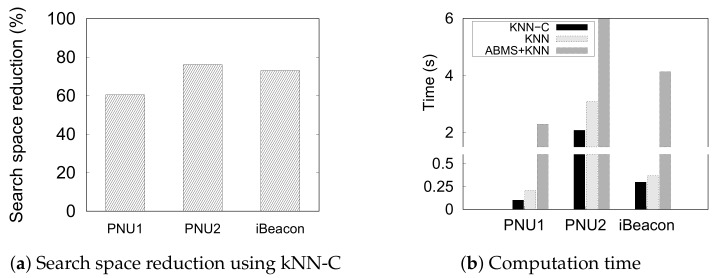
Efficiency of kNN-based methods to predict a semantic position.

Figure 11 presents the efficiency of the three kNN-based methods, including the reduced search space in each dataset. In this experiment, we fix the number of neighbors (*k*) to 10 to estimate a semantic position. With the efficiency, we confirm the feasibility of each method in a real-time. It is evident that ABMS + kNN consumes the longest computation time to perform an estimation, followed by a significantly shorter time for kNN, and the shortest time for kNN-C. The search space reduction by the constraints (Figure 11a) highly influences the computation time of kNN-C (Figure 11b). The computation time largely decreases from the original kNN because kNN-C reduces the reference set/search space size owing to the movement constraints from the previously inferred position. In a current inference of position ${\hat{s}}^{t}$, kNN-C only looks for the neighboring semantic position from the previous position ${\hat{s}}^{t-1}$ instead of observing all instances of the reference set. ABMS + kNN performed poorly because it performs kNN until the inferred position does not change or converges. The prediction time of ABMS + kNN is nearly 10× that of kNN. Even in the largest dataset, PNU2, it needs more than 6 s to predict a single position. Thus, We can conclude that ABMS + kNN is not suitable for real-time inference due to the long prediction time. This result shows that the constraints significantly reduce the cost of computation for indoor positioning.

Table 5 depicts the comparison of the kNN methods based on the change of *k*. It is evident that, by using a smaller value of *k*, kNN-C achieves the best result. Although, with a larger value of *k* in dataset PNU1 and PNU2, both kNN and ABMS + kNN outperform kNN-C. This underperformance is mainly because the number of neighbor affects the performance of kNN significantly on the constrained ones.

In kNN-based methods, if we consider more neighbors for the indoor positioning, we are more prone to irrelevant references. Using the constraints, the number of relevant references are reduced significantly because the references were divided into subsets of the neighboring areas of the previous estimated position. Considering a fewer number of neighbors is slightly beneficial because it saves time to infer a semantic position as it considers a fewer number of references practically.

#### 5.4.4. Deep Neural Network

We used simple Deep Neural Networks (DNN) as the other ML-based indoor positioning method. We compared the performance of constrained and original DNN in terms of SPER. We averaged the results from the 10 repeated experiments for each parameter (hidden layers and datasets).

**Figure 12:**
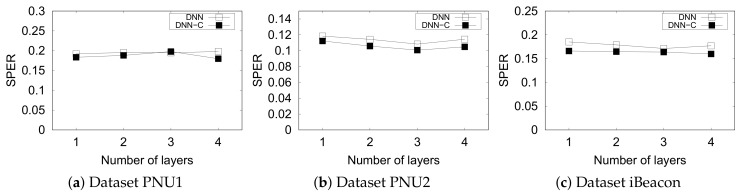
Performance of DNN and DNN-C with various hidden layers.

Figure 12 shows that the constraints improve the SPER on nearly all datasets. However, the original DNN outperformed DNN-C on the PNU1 dataset when their layers are three. On the other hand, apparently, the number of hidden layers of DNN does not relate to the performance of SPER.

#### 5.4.5. Error vs. SPER

The error and SPER have slightly different effectiveness measures in indoor semantic positioning. As described before, error does not consider the distance between the predicted semantic position ${\hat{s}}^{\left(t\right)}$ and the ground truth $s^{\left(t\right)}$ at time *t*. We fixed the parameters of each method according to the default value in Table 4 (HMM window length = 3, *k* = 10, and DNN hidden layers = 4).

**Figure 13:**
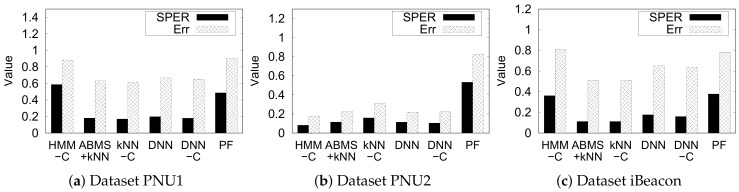
Comparison of SPER and error for each approach.

Figure 13 presents the error and the SPER of all studied methods, except the naive kNN and the unsupervised multivariate HMM for brevity. It is evident that, in a particular method and dataset, error and SPER do not have a linear relationship. This relationship is possible because SPER highly depends on the maximum distance from a single semantic position in the semantic graph, which varies in different indoor environments. The longer the longest distance in a semantic graph, the more likely SPER is lower. In contrast, error does not depend on any characteristic of the semantic graph.

**Figure 14:**
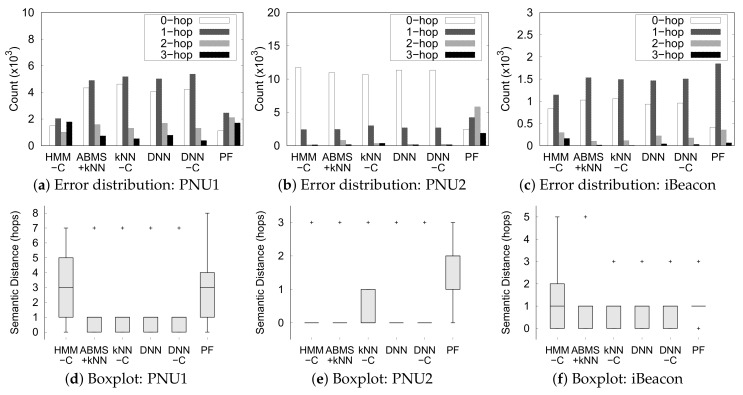
Error analysis of the indoor positioning predictions and the ground truth.

The error analysis in Figure 14 shows more details about the performance of each method and the reason for using SPER instead of error. Figure 14a–c shows the count distribution of the hops needed to travel from the correct semantic position to the predicted semantic position for each dataset in the semantic graph (semantic distance). We only show the count of the four smallest distances in the figure because the number of ground truths and predicted semantic position pairs gets significantly smaller for the larger distance. We see that ML-based methods mispredict the majority of the semantic position with a distance of 1 hop in all datasets, except for HMM-C in dataset PNU1. If we use only error, we cannot capture this detail because error only considers the count of a 0-hop distance of the ground truth and predicted positions. Figure 14d–f shows boxplot of the semantic distance from the correct semantic position to the predicted semantic position for each dataset. The boxplots reflect similar insights to the error distribution for the majority of the misprediction with a 1-hop distance. The indoor positioning techniques still predict a small amount of positions that are far from ground truth as outliers, except for the particle filter in PNU1 and PNU2 dataset. Thus, it is evident that the particle filter performed poorly in every dataset.

Furthermore, most of the methods perform well in the PNU2 dataset. This is plausible because PNU2 has a larger dataset for training and a simpler semantic graph than the other two datasets. In contrast, HMM performs generally worse than the other methods, except in PNU2. This means that the HMM approach may not perform well in a smaller dataset and more complex setting. Also, most of the methods outperformed particle filter in dataset PNU2. From the dataset description in Table 3, PNU2 has more observation points than the PNU1 and iBeacon data. In other words, on average, a trajectory in dataset PNU2 is longer (has more points) than a trajectory in datasets PNU1 and iBeacon. Thus, we can conclude that the particle filter method performed worse than ML-based indoor positioning for longer trajectories.

#### 5.4.6. Validity

**Figure 15:**
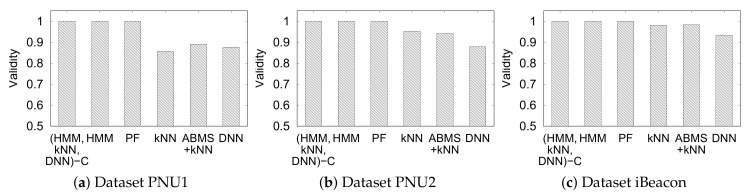
Validity of indoor positioning with constraints and the other method.

Figure 15 shows the validity of the unconstrained and constrained approaches, including the unsupervised MHMM (HMM). Evidently, the constrained approaches extract the valid trajectory (always 1) owing to the movement constraints.

The movement constraints ensures the validity to be 1 because the constraints restricts the inferred positions to be close to each other. The HMM and particle filter also extract valid trajectories. HMM always extract valid trajectories as it is the more optimal form of the HMM with constraints. Meanwhile, the particle filter infers the positions by simulating the user movements by the parameter of walking speed. Thus, the particle filter by default provides valid trajectories.

It can also be observed that the unconstrained approach yields invalid trajectories but with validity in the range of (0.85–1]. Hence, the distance of the consecutive predicted semantic position by the unconstrained approach is not too far.

#### 5.4.7. Dynamic Environment

**Figure 16:**
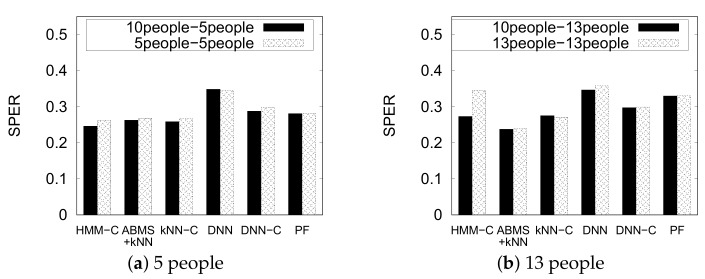
Results of indoor positioning in a dynamic environment.

Figure 16 shows the performance of the indoor positioning methods on a dynamically changing environment using dataset PNU3. We omit the original kNN and HMM again for brevity. We denote each result as (training)-(test) notation. For example, in the chart, the “10people-5people” result means we train the model using the 10 people setting and test the model using the 5 people setting. Particle filter gives exactly equal results because it does not train any model and works directly on the test. Interestingly, most of the methods give slightly similar or improved results of the dynamic setting (10 people-5 people and 10 people-13 people) to its original setting counterpart (5 people-5 people and 13 people-13 people), except for HMM-C that significantly improves the performance of the 13 people setting using the 10 people dataset. We can infer that a carefully selected environment as training set can improve the performance of the indoor positioning of a dynamic environment.

On the other hand, we see that the DNN without constraint does not work well on both settings. However, the DNN with constraint approach significantly improves the result. Meanwhile, the particle filter works well in the 5-people setting but it is slightly outperformed by the ML-based methods. The underperformance of particle filter is clearly shown in the 13-people setting. The ABMS + kNN method outperformed kNN with constraint in the 13-people setting, whereas they show a similar result in the 5-people setting. However, note that ABMS + kNN does not always give valid results like kNN with constraint and requires more processing time.

#### 5.4.8. Discussion

Our experiments show that, among different indoor positioning methods, the application of movement constraints yields valid semantic trajectory extraction. Ensuring the validity of the trajectory may or may not affect the correctness of the results, represented by SPER and error. For example, the result of ABMS + kNN is better than kNN-C in the PNU2 dataset but it produces a less valid trajectory.

Even though particle filter, as non-ML-based indoor positioning, gives valid trajectory, it underperforms the ML-based indoor positioning results in almost every dataset. In addition, the particle filter requires heavy computation due to the resampling technique.

In contrast, the constraints in DNN improved the correctness of the indoor positioning. A perfect indoor trajectory estimation (SPER and error = 0) should provide a valid trajectory. However, this erroneous estimation is still inevitable due to the obstructions in indoor environment. Thus, a better RSSI data quality enhancement would improve the quality of the indoor positioning. Note that the RSSI data enhancement effort should not burden the computation to work in real time. On the other hand, we show that our approaches worked in different types of indoor environments, considering the various numbers of installed beacons, semantic positions, indoor layouts, movement constraints, and the number of people in the indoor space.

## 6. Conclusions

In this paper, we presented movement constraints to extract valid indoor semantic trajectories using BLE beacons. We extended some indoor positioning techniques using the proposed movement constraints to prevent the prediction of any semantic position distant from the previous estimation, thereby resulting in an invalid trajectory. We conducted the comprehensive experiments of four different indoor settings.

Our experiments demonstrated that the proposed movement constraint-based approaches extract valid trajectories that are comparable to the unconstrained and non-ML approaches. On the other hand, we also show that our proposed approach can handle a dynamic indoor environment.

For all approaches, the proposed methods with constraints yielded a comparable positioning quality with respect to their non-constrained approaches. The online HMM with constraints provides a slightly similar performance to its original counterpart. For kNN, the movement constraint, in addition to improving the correctness, also increased the efficiency by 60–70% and the speed by 1.5 times. Likewise, the constraints also improved the DNN in both correctness and validity.

In the future, we plan to improve the quality of RSSI data from the beacons and to discover interesting patterns from the extracted semantic indoor trajectory.

## Figures and Tables

**Table 1:** HMM of semantic trajectory extraction.

Symbol	Description
*S*	Set of semantic positions *S*
*O*	Set of observations of each beacon RSSI (received strength signal indicator) value
*A*	Movement probability between each semantic position $A\left(i,j\right)=P\left(s_{i}\to s_{j}\right)\;$ where $\;s_{i},s_{j}\in S$
*B*	Probability of beacon *m* emitting RSSI *k* at semantic position $s_{i}\in S$: $B\left(i,m,k\right)=P\left(s_{i}|O_{m,k}\right)$
π	Occuring probability of semantic position at t=1

**Table 2:** Studied approaches for the experiments.

Approach Name	Abbreviation
Naïve k-NN	kNN
**k-NN with constraint**	**kNN-C**
Adaptive Bandwidth Mean Shift + k-NN [8]	ABMS + kNN
Unsupervised Multivariate Hidden Markov Model	MHMM
**MHMM with online Viterbi and constraint**	**HMM-C**
Deep Neural Network	DNN
**Deep Neural Network with constraint**	**DNN-C**
Particle Filter	PF

**Table 3:** Detailed dataset description.

Dataset	PNU1	PNU2	PNU3	iBeacon
Deployed beacons	8	10	18	13
Semantic positions	14	7	8	48
Area	64.5 m × 17.4 m	64.5 m × 17.4 m	18 m × 17.4 m	80 m × 50 m
Reference set	72	10	**44 (10 people)**	143
			21 (5 people)	
			49 (13 people)	
Test set	28	4	6 (5 people)	16
			12 (13 people)	
Total length	38,967	125,883	74,238	1420

**Table 4:** Parameter setup (the default values are in bold).

Parameter	Setup
HMM window	2, **3**, 4, 5, 6
k	1, 5, **10**,15, 20
DNN hidden layer	1 (48)
	2 (48, 80)
	3 (48, 80, 92)
	**4 (48, 80, 92, 64)**

**Table 5:** Comparison of the performance of kNN methods based on Semantic Positioning Error Rate (SPER).

Dataset	Method	Number of Neighbors (k)				
		1	5	10	15	20
PNU1	kNN-C	0.208	0.204	0.170	0.268	0.282
	kNN	0.236	0.221	0.216	0.197	0.196
	ABMS + kNN	0.220	0.192	0.181	0.177	0.178
PNU2	kNN-C	0.115	0.149	0.159	0.166	0.128
	kNN	0.159	0.123	0.113	0.110	0.120
	ABMS + kNN	0.136	0.112	0.115	0.118	0.121
iBeacon	kNN-C	0.100	0.110	0.112	0.113	0.114
	kNN	0.104	0.113	0.118	0.117	0.120
	ABMS + kNN	0.108	0.121	0.122	0.118	0.122
